# How parenting styles affect primary school students’ subjective well-being? The mediating role of self-concept and emotional intelligence

**DOI:** 10.3389/fpsyg.2024.1425777

**Published:** 2024-10-22

**Authors:** Tiantian Gao, Baoqian Li, Shuxian Liang, Wanmeng Zhou, Xinyi Zhu, Lu Bai, Guoqiang Li

**Affiliations:** Jing Hengyi School of Education, Hangzhou Normal University, Hangzhou, China

**Keywords:** primary school students, parenting styles, subjective well-being, self-concept, emotional intelligence, mediating role

## Abstract

**Purpose:**

To explore the relationship between parenting styles and self-concept, emotional intelligence, and subjective well-being among primary school students.

**Methods:**

In total, 1,683 students from 10 primary schools in Hangzhou, China, were surveyed using a simple random sampling method. Participants completed the Parenting Style Scale, the Self-Concept Scale, the Emotional Intelligence Scale, and the Subjective Well-Being Scale.

**Results:**

(1) Father’s and mother’s emotional warmth was significantly positively correlated with students’ subjective well-being (*r* = 0.513, *p* < 0.01 and *r* = 0.478, *p* < 0.01, respectively). Father’s and mother’s rejection was significantly negatively correlated with students’ subjective well-being (*r* = −0.257, *p* < 0.01 and *r* = −0.285, *p* < 0.01, respectively). Father’s overprotection was significantly negatively correlated with students’ subjective well-being (*r* = −0.178, *p* < 0.01; *r* = −0.227, *p* < 0.01). (2) Self-concept and emotional intelligence acted as chain mediators between father’s and mother’s emotional warmth and students’ subjective well-being (0.337, 0.477 and 0.366, 0.520, respectively). Self-concept and emotional intelligence acted as chain mediators between father’s and mother’s rejection and students’ subjective well-being (−0.590, −0.377 and −0.693, −0.460, respectively). Self-concept chain-mediated between father’s and mother’s overprotection and students’ subjective well-being (−0.380, −0.224 and −0.413, −0.264 respectively).

**Conclusion:**

Parenting styles can affect primary school student’s subjective well-being both directly and through mediating effects. Self-concept and emotional intelligence partially mediate the impact of emotional warmth and rejection parenting styles, and fully mediate the impact of father’s overprotective parenting on subjective well-being.

## 1 Introduction

With the development of psychology and family education, an increasing number of researchers have studied subjective well-being (Diener et al., 2018; Deng et al., 2015). Parents have also begun to consider their children’s mental health, and are more inclined to determine whether their children really feel happy than solely focus on their academic achievements. Primary school plays a crucial role in the development of an individual’s personality, motivation, and life satisfaction (García et al., 2006; Lari, 2023) as these students are faced with dramatic psychological and physiological changes. In China, the rapid development of society means students are under tremendous pressure and often suffer from psychological problems, such as anxiety, social disorders, and interpersonal disorders (Ebrahimi et al., 2017). To address this situation, China recently enacted a policy of burden reduction, which advocates reducing students’ schoolwork pressure and improving students’ sense of well-being.

The ecosystem theory suggests that the family, as the most intimate microsystem of individual development, has an impact on all aspects of individual development (Bronfenbrenner, 1986). Parenting style means “the educational concepts, attitudes and all the words and actions of the parents in the upbringing of their children” (Darling and Steinberg, 1993). It has been shown that there is a significant correlation between different parenting styles and subjective well-being among primary school students (Huang et al., 2024; Furnham and Cheng, 2000). Negative parenting styles negatively predicted elementary school students’ subjective well-being, and positive parenting styles positively predicted primary these students’ subjective well-being. Although the relationship between parenting styles and subjective well-being has been corroborated, the underlying influencing mechanisms remain to be explored.

### 1.1 Mediating role of self-concept

Self-concept refers to the self-perception developed through experience and understanding of that experience. For example, an individual’s self-perception derives from their experiential experiences of interpersonal interactions, self-attributes, and the social environment (Cooper et al., 2018; Shavelson and Bolus, 1982). Attachment theory posits that if parents are caring, understanding, and supportive in their parenting behaviors, then it is beneficial for children to form secure attachments and develop positive self-beliefs (Bretherton and Munholland, 1999). For example, the child believes that they are lovable and valuable. On the contrary, children may develop negative perceptions such as incompetence and worthlessness in the absence of such care and support. It has been shown that positive parenting styles are conducive to students developing a higher self-concept, whereas negative parenting styles tend to lead to a lower self-concept in students (Heymann, 2000; McClun and Merrell, 1998). A revalidation study by Martinez et al. (2020) drew the same conclusions.

In addition, a child’s positive self-concept impacts their long-term subjective well-being. A positive self-concept represents a psychological force that can overcome or negate adverse environmental and risk factors that may hinder healthy growth and well-being (Leflot et al., 2010; Marsh, 1990). It has also been shown that people’s well-being mainly depends on their self-perception (Calero et al., 2018). When self-identity is well developed, students can recognize themselves, view themselves, accept themselves dialectically and objectively, and improve their ability to adapt and regulate, thereby enhancing their subjective well-being. The experience of negative affect means students are more likely to doubt themselves and be unsure about their status and future planning.

### 1.2 Mediating role of emotional intelligence

Emotional intelligence refers to “the ability to recognize the intrinsic meaning of emotions, to use knowledge and skills to identify problems, to solve practical problems through analysis and logical reasoning, and to use emotions to facilitate cognition” (Salovey et al., 2003). Parenting styles are an essential factor in the development of children’s emotional intelligence; authoritative parenting styles can lead to a positive assessment of emotional intelligence, whereas authoritarian parenting styles may hinder the nurturing and development of children’s emotional intelligence (Argyriou et al., 2016; Mortazavizadeh et al., 2022). Emotions reflected in the family affect children’s perception of and ability to regulate emotions. Therefore, children with supportive parents have better emotional management skills and children who grow up in a loving parental environment have higher levels of emotional intelligence than those without such environments (Lopes et al., 2003). Many national studies have shown that parenting styles and attitudes toward children can positively predict children’s emotional development (Yating et al., 2010). In contrast, negative parenting styles can seriously hinder the development of students’ mental and emotional intelligence (Yating et al., 2010).

In addition, there is a positive association between emotional intelligence and well-being (Jung et al., 2019). Students with high emotional intelligence have a better emotional state and a greater sense of well-being and fulfillment than those with low emotional intelligence (Sánchez-Álvarez et al., 2015). Furthermore, emotional intelligence is a strong determinant of self-esteem, and improving emotional intelligence is important in strengthening the foundation of self-esteem in adolescents (Cheung et al., 2015).

### 1.3 Relationship between self-concept and emotional intelligence

Children’s emotional intelligence is positively related to their psychological adjustment, intrinsic motivation, and academic achievement, and negatively related to social anxiety (Cobos-Sánchez et al., 2017; Cejudo et al., 2018). Individuals with high emotional intelligence are likely to be psychologically healthier and more likely to achieve well-being than those with low emotional intelligence. They are also more able to extrapolate their perception, understanding, and management skills to the emotions of others, which leads to more satisfying interpersonal relationships and self-concept (Ciarrochi et al., 2000; Martínez-Monteagudo et al., 2021). One study suggested emotional intelligence can be used to enhance subjective well-being (Chen et al., 2016).

Self-concept also has a stimulative effect on emotional intelligence. For individuals in the medium to high range of self-concept clarity, higher emotional processing skills should contribute to their self-reported ability to evaluate, manage, and regulate the emotions of others (Light, 2017). High self-concept clarity brings a high degree of transparency and a subtle understanding of one’s emotions (Merdin-Uygur et al., 2018; Hanley and Garland, 2017). People with higher emotional clarity tend to be better communicators and deal with conflict more effectively than those with lower emotional clarity (Fitness, 2001; Xiang et al., 2023).

### 1.4 Present study and hypotheses

**Figure 1:**
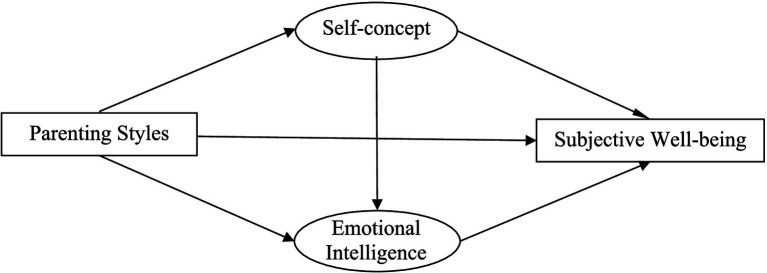
A Hypothetical model of the influence of parenting styles on subjective well-being.

Previous research has revealed relationships between students’ subjective well-being, parenting styles, self-concept, and emotional intelligence. Studies that examined parenting styles and emotional intelligence as separate factors influencing subjective well-being drew separate conclusions, but no systematic study has examined the relationship between the three factors (Ebrahimi et al., 2017; Martínez-Monteagudo et al., 2021). The purpose of this study was to explore the relationship between parenting styles and subjective well-being based on the addition of two mediating variables: self-concept and emotional intelligence. We explored whether these variables mediated the relationship between parenting styles and students’ subjective well-being. This allowed us to investigate detailed factors that influenced the subjective well-being of elementary school students, which will provide a theoretical basis for enhancing subjective well-being in this population as well as schools’ mental health education. Therefore, this study examined the mediating roles of self-concept and emotional intelligence in the relationship between parenting styles and subjective well-being. We proposed the following hypotheses (hypothesized model is shown in Figure 1).

H1: Parenting styles directly affect the subjective well-being of primary school students.

H2: Self-concept mediates the relationship between parenting styles and the subjective well-being of primary school students.

H3: Emotional intelligence mediates the relationship between parenting styles and the subjective well-being of primary school students.

H4: Self-concept and emotional intelligence play multiple mediating roles in the relationship between parenting styles and subjective well-being.

## 2 Design

### 2.1 Participants and context

This study was conducted in October 2022 in primary schools in Hangzhou, China. We selected 40 grades (10 classes per grade) across 10 schools as survey respondents using a simple random sampling method. These respondents were all in grades 3–6. The questionnaires took 20 min to complete and were administered on-site. We collected 1,810 completed questionnaires for this survey (Table 1). After excluding questionnaires with obvious irregularities and inconsistencies in the answers, there were 1,683 valid questionnaires (92.98%), of which 878 (52.2%) were from male students and 805 (47.8%) were from female students. There were 1,337 (79.4%) urban students and 346 (20.6%) rural students. In total, 597 (35.5%) students were from one-child families and 1,086 (64.5%) were from non-one-child families. Students’ were aged 9–12 years, with a mean age of 10.36 ± 1.13 years. The design and methodology of our study complied with the requirements of the Ethics Committee of Hangzhou Normal University. Ethical approval was obtained before conducting this study, and written parental consent was obtained before the survey. The questionnaires were administered and collected with the consent of the participants and schools.

**Table 1:** Demographic characteristics of primary students (*N* = 1,683).

Characteristic		*N* = 1,683	Percent (%)
Gender, *n* (%)			
	Male	878	52.2%
	Female	805	47.8%
Age, M (SD)	10.360 (1.130)		
Home location			
	Town	1,337	79.4%
	Village	346	20.6%
Family composition			
	Both parents raising	1,544	91.7%
	Single parent raising	126	7.5%
	Foster parents	13	0.8%
Only child in family			
	Yes	597	35.5%
	No	1,086	64.5%

M = Mean; SD=Standard deviation.

### 2.2 Methods

#### 2.2.1 Parenting style scale

This study used the Short Form Parenting Style Questionnaire (s-EMBU-c). This is a revised version of the s-EMBU, which was compiled by domestic scholar Jiang and foreign researcher Arrindell, and reflected the local conditions of China (Arrindell and Engebretsen, 2000). The scale has two subscales (fathers and mothers) and covers three dimensions: emotional warmth, rejection, and overprotection. The scale includes 21 questions for each subscale: seven questions on emotional warmth, six questions on rejection, and eight questions on overprotection. The scale has four response options from negative (“Never”) to positive (“Always”). This study confirmed that the scale had good reliability and validity. The reliability of the father’s and mother’s parenting styles subscales were 0.815 and 0.813, respectively. The validity indices for the father’s parenting style subscale were: *X*^2^/df = 4.832, goodness of fit index (GFI) = 0.953, Tucker-Lewis index (TLI) = 0.899, comparative fit index (CFI) = 0.911, and root mean square error of approximation (RMSEA) = 0.046. The validity indices for the mother’s parenting style subscale were: *X*^2^/df = 3.812, GFI = 0.963, TLI = 0.912, RMSEA = 0.922 and RMSEA = 0.040.

#### 2.2.2 Self-Concept Scale

The instrument used to measure self-concept in this study was the Self-Concept Scale. This scale was revised by Guotao Zhou and Lingfeng He, based on the scale compiled by Song and Hattie (Guotao and Lingfeng, 1996; Song and Hattie, 1984). The scale uses a five-level scoring method and contains seven dimensions: ability-self, achievement-self, class-self, family-self, companion-self, body-self, and confidence-self. Each subscale comprises five items, giving a total of 35 items. The seven dimensions are grouped on three subscales: academic self-subscale (ability, achievement, and class), social self-subscale (family and peers), and self-presentation subscale (body and self-confidence). This study confirmed that the scale had good reliability and validity. The test–retest reliability of the scale was 0.83, the correlation with Marsh’s Self-Description Questionnaire was 0.922, and the validity indices were: *X*^2^/df = 5.436, GFI = 0.862, TLI = 0.864, CFI = 0.873, and RMSEA = 0.050.

#### 2.2.3 Emotional Intelligence Scale

The Emotional Intelligence Scale used in this study was based on Goleman’s emotional intelligence theory (Goleman, 1995). It has five dimensions: awareness of own emotions, proper management of emotions, self-motivation, awareness of others’ emotions, and management of others’ emotions in interpersonal situations. The scale has 23 questions and is scored on a 5-point Likert scale from 1 = “not at all” to 5 = “completely.” A higher score indicates higher emotional intelligence. As the scale’s reliability was based on data from Taiwan, the font, grammar, and vocabulary were slightly modified to conform to use in Mainland China, but no changes were made to the structure of the scale or the content of the items (Yafang, 2005). This study confirmed that the scale had good reliability and validity. The Cronbach’s *α* for the total scale was 0.93, and the validity indices were: *X*^2^/df = 6.004, GFI = 0.938, TLI = 0.942, CFI = 0.949, and RMSEA = 0.053.

#### 2.2.4 Subjective well-being scale

This study used Xinggui Zhang’s MSLSS (Multidimensional Students’ Life Satisfaction Scale), which was based on Hunbner’s MSLSS, to develop a Well-being Scale specifically for Chinese adolescent school students (Huebner and Alderman, 1993; Xinggui, 2003). The scale included an adolescent student life satisfaction scale and a sense of happiness scale; life satisfaction covered both overall life satisfaction and domain-specific life satisfaction (family satisfaction, friendship satisfaction, academic satisfaction, school satisfaction, freedom satisfaction, and environment satisfaction). The scale has 36 items; seven items on friendship and family satisfaction, six items on school and academic satisfaction, and five items on freedom and the environment. We used the 7-point rating system for this scale. Except for items 3, 4, 9, and 10, all questions were scored in the same direction. The scale comprises positive and negative emotions and reports the feelings experienced in the past week. The 14 items included six positive emotion items and eight negative emotion items that are scored using a 5-point system. This study confirmed that this scale had good reliability and validity. The homogeneity reliability of the scale ranged from 0.07 to 0.091, and the stability reliability of the scale and its subscales ranged from 0.54 to 0.85. The Cronbach’s *α* for the total scale was 0.918, and the validity indices were: *X*^2^/df = 5.436, GFI = 0.862, TLI = 0.864, CFI = 0.873, and RMSEA = 0.050.

### 2.3 Data analysis

First, we coded the questionnaire data after excluding questionnaires with obvious regular responses as well as those with obvious inconsistencies in the answers. The coded data were examined using descriptive statistics and underwent correlation analysis using SPSS 24.0. AMOS 24.0 software was used for validation factor analysis. Next, we established the chain mediation model. The mediation effect test was conducted using a bootstrap method with a sampling number of 5,000; when the 95% confidence interval did not include 0, it indicated a mediating effect.

## 3 Results

### 3.1 Descriptive and correlation analyses

Table 2 shows the means and standard deviations for parenting styles, self-concept, emotional intelligence, and subjective well-being, and their correlations. Regarding correlations, subjective well-being was positively correlated with the parenting style of emotional warmth. Emotional intelligence, self-concept, and subjective well-being were negatively correlated with the rejection and overprotection parenting styles. Self-concept was negatively correlated with the rejection and overprotection parenting styles.

**Table 2:** Descriptive statistics for each variable (*N* = 1,683).

		M ± SD	1.		
			(1)	(2)	(3)
1.Fathers’ parenting styles	(1) Father’s emotional warmth	25.46 ± 6.75	–		
	(2) Father’s Rejection	8.82 ± 3.63		–	
	(3) Father’s overprotection	16.10 ± 4.79			–
2. Self-concept		125.89 ± 23.69	0.525^**^	−0.274^**^	−0.225^**^
3. Emotional intelligence		91.09 ± 17.32	0.441^**^	−0.194^**^	−0.165^**^
4. Subjective well-being		181.14 ± 27.03	0.513^**^	−0.257^**^	−0.178^**^
1.Mothers’ Parenting Style	(1) Mother’s emotional warmth	26.70 ± 6.22	–		
	(2) Mother’s rejection	8.82 ± 3.48		–	
	(3) Mother’s overprotection	17.22 ± 5.21			–
2. Self-concept		125.89 ± 23.69	0.504^**^	−0.311^**^	−0.275^**^
3. Emotional Intelligence		91.09 ± 17.32	0.418^**^	−0.209^**^	−0.188^**^
4. Subjective Well-being		181.14 ± 27.03	0.478^**^	−0.285^**^	−0.227^**^

M = Mean; SD=Standard deviation; ^**^p < 0.01.

### 3.2 Bootstrap analysis of mediating effect significance test

Parenting styles were categorized into three dimensions: emotional warmth, rejection, and overprotection. Three mediation models were developed to test the mediation effect of the relationship between the parenting style and subjective well-being for each dimension.

#### 3.2.1 Mediating role of self-concept and emotional intelligence between the emotional warmth parenting style and subjective well-being

Regression analysis showed that parental warmth positively predicted students’ self-concept, emotional intelligence, and subjective well-being. Self-concept significantly positively predicted subjective well-being and emotional intelligence. Emotional intelligence significantly positively predicted subjective well-being (Tables 3, 4).

**Table 3:** Regression analysis of the relationship between father’s emotional warmth variables.

Regression equation		Global FIT index		Significance of regression coefficient	
Result variable	Predictive variables	*R* ^2^	*F*	*β*	*t*
Self-concept	Gender			0.0404	2.0087
	Age	0.2795	231.1178	0.0179	0.8782
	Father’s emotional warmth			0.5227	26.0153
Emotional intelligence	Gender	0.4189	321.9084	0.0859	4.7504
	Age			0.0372	2.0561
	Self-concept			0.5377	25.3015
	Father’s emotional warmth			0.1567	7.3952
Subjective well-being	Gender	0.5950	524.3737	−0.0126	−0.8319
	Age			−0.0179	−1.1849
	Emotional intelligence			0.3604	18.2396
	Self-concept			0.3990	19.2902
	Father’s emotional warmth			0.1458	8.1127

**Table 4:** Regression analysis of the relationship between mother’s emotional warmth variables.

Regression equation		Global FIT index		Significance of regression coefficient	
Result variable	Predictive variables	*R* ^2^	*F*	*β*	*t*
Self-concept	Gender	0.2595	208.7586	0.0546	2.6799
	Age			0.0543	2.6686
	Mother’s emotional warmth			0.5029	24.7016
Emotional intelligence	Gender	0.416	318.0019	0.0858	4.735
	Age			0.0408	2.2501
	Self-concept			0.5487	26.1081
	Mother’s emotional warmth			0.141	6.7328
Subjective well-being	Gender	0.5899	513.4785	−0.0151	−0.8895
	Age			−0.0136	−0.9945
	Emotional intelligence			0.3676	18.535
	Self-concept			0.4122	19.9079
	Mother’s emotional warmth			0.1165	6.5526

The results showed that self-concept and emotional intelligence completely mediated the relationship between father’s emotional warmth and students’ subjective well-being. The total mediating effect was 1.4662. The mediating pathways and effect values were: father’s emotional warmth → self-concept → subjective well-being (0.837); father’s emotional warmth → emotional intelligence → subjective well-being (0.2261); and father’s emotional warmth → self-concept → emotional intelligence → subjective well-being (0.4054). The 95% confidence intervals for the three paths did not contain 0, indicating that the indirect effects of the two mediating variables were significant (Table 5).

**Table 5:** An analysis of the chain-mediated effects of self-concept and emotional intelligence on the relationship between fathers’ emotional warmth and subjective well-being.

	Effect value	BootSE	BootLLCI	BootULCI
Total effect	2.0497	0.0812	1.8905	2.2088
Direct effect	0.5834	0.0719	0.4424	0.7245
Mediating effect	1.4662	0.0666	1.3372	1.5997
FEW-SC-SWB	0.8347	0.0589	0.72	0.9481
FEW-EI-SWB	0.2261	0.0366	0.1564	0.3005
FEW-SC-EI-SWB	0.4054	0.0358	0.3369	0.4764

FEW=Father’s emotional warmth; SC=Self-concept; SWB=Subjective well-being; EI = Emotional intelligence.

Self-concept and emotional intelligence played complete mediating roles between mother’s emotional warmth and subjective well-being, with a comprehensive summary effect value of 1.5663. The mediating pathways and effect values were: mother’s emotional warmth → self-concept → subjective well-being (0.9005); mother’s emotional warmth → emotional intelligence → subjective well-being (0.2252); and mother’s emotional warmth → self-concept → emotional intelligence → subjective well-being (0.4406). The 95% confidence intervals for these three paths did not contain 0, indicating that the indirect effects of both mediating variables were significant (Table 6).

**Table 6:** An analysis of the chain-mediated effects of self-concept and emotional intelligence on the relationship between mothers’ emotional warmth and subjective well-being.

	Effect value	BootSE	BootLLCI	BootULCI
Total effect	2.0724	0.0901	1.8957	2.249
Direct effect	0.5061	0.0772	0.3546	0.6576
Mediating effect	1.5663	0.0763	1.4161	1.7177
MEW-SC-SWB	0.9005	0.0648	0.7726	1.0296
MEW-EI-SWB	0.2252	0.0408	0.149	0.3096
MEW-SC-EI-SWB	0.4406	0.0393	0.3657	0.5198

MEW = Mother’s emotional warmth; SC=Self-concept; SWB=Subjective well-being; EI = Emotional intelligence.

**Figure 2:**
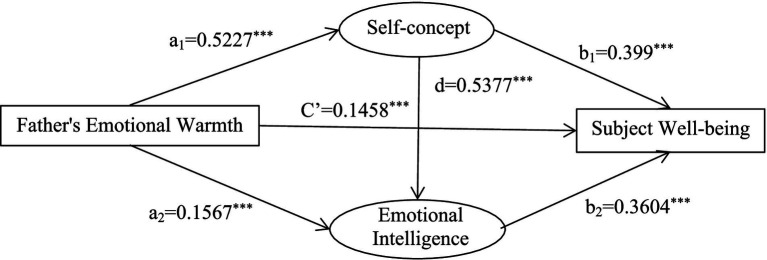
A chain-mediated model of fathers’ emotional warmth, self-concept, emotional intelligence and subjective well-being.

**Figure 3:**
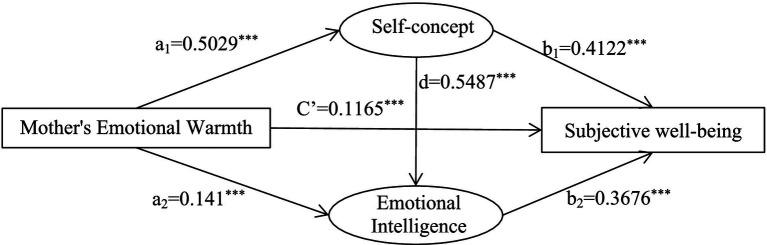
A chain-mediated model of mothers’ emotional warmth, self-concept, emotional intelligence and subjective well-being.

The emotional warmth of fathers and mothers were used as independent variables, self-concept and emotional intelligence as mediating variables, and subjective well-being as the dependent variable to establish a chain-mediated model (Figures 2, 3).

#### 3.2.2 Mediating role of self-concept and emotional intelligence between the rejection parenting style and subjective well-being

Regression analysis showed that parental rejection negatively predicted self-concept, emotional intelligence, and subjective well-being. Self-concept positively predicted emotional intelligence and executive well-being, and emotional intelligence positively predicted subjective well-being (Tables 7, 8).

**Table 7:** Regression analysis of the relationship between father’s rejection variables.

Regression equation		Global FIT index		Significance of regression coefficient	
Result variable	Predictive variables	*R^2^*	*F*	*β*	*t*
Self-concept	Gender	0.0778	50.2419	0.0466	2.0489
	Age			0.0543	2.6686
	Father’s rejection			−0.2685	−11.7384
Emotional intelligence	Gender	0.4014	299.3516	0.0814	4.4141
	Age			0.0373	2.0298
	Self-concept			0.6159	32.3063
	Father’s rejection			−0.0155	−0.8094
Subjective well-being	Gender	0.5838	500.7358	−0.0237	−1.5343
	Age			−0.0201	−1.3147
	Emotional intelligence			0.3866	19.5866
	Self-concept			0.4423	22.0992
	Father’s rejection			−0.0642	−4.0222

**Table 8:** Regression analysis of the relationship between mother’s rejection variables.

Regression equation		Global FIT index		Significance of regression coefficient	
Result variable	Predictive variables	*R* ^2^	*F*	*β*	*t*
Self-concept	Gender	0.101	66.9364	0.0399	1.7758
	Age			0.0533	2.3755
	Mother’s rejection			−0.3078	−13.6945
Emotional intelligence	Gender	0.4012	299.2199	0.0824	4.4839
	Age			0.0377	2.0548
	Self-concept			0.6166	31.9281
	Mother’s rejection			−0.0112	−0.5828
Subjective well-being	Gender	0.5844	502.0144	−0.0207	−1.345
	Age			−0.0182	−1.1919
	Emotional intelligence			0.3869	19.6201
	Self-concept			0.4377	21.6997
	Mother’s rejection			−0.0698	−4.3432

Self-concept and emotional intelligence partially mediated parenting styles and subjective well-being. The mediating effect was −1.4065. The mediating pathways and effect values were: father’s rejection → self-concept → subjective well-being (−0.8853); father’s rejection → emotional intelligence → subjective well-being (−0.0446); and father’s rejection → self-concept → emotional intelligence → subjective well-being (−0.4766). The path of father’s rejection → emotional intelligence → subjective well-being contained 0 in the 95% confidence interval, which indicated that the indirect effect of emotional intelligence was not significant. The other two paths did not contain 0 in the 95% confidence interval, indicating that the indirect effects of both mediating variables were significant (Table 9).

**Table 9:** An analysis of the chain-mediated effects of self-concept and emotional intelligence on the relationship between father’s rejection and subjective well-being.

	Effect value	BootSE	BootLLCI	BootULCI
Total effect	−1.8851	0.1715	−2.2214	−1.5488
Direct effect	−0.4786	0.119	−0.7119	−0.2452
Mediating effect	−1.4065	0.1352	−1.6824	−1.1535
FR-SC-SWB	−0.8853	0.092	−1.0696	−0.7097
FR-EI- SWB	−0.0446	0.062	−0.1695	0.0766
FR-SC-EI-SWB	−0.4766	0.055	−0.59	−0.3769

FR = Father’s rejection; SC=Self-concept; SWB=Subjective well-being; EI = Emotional intelligence.

The analysis of mediating effects showed that self-concept and emotional intelligence played a partial mediating role in mother’s rejection and subjective well-being. The total mediating effect value was −1.6542. The mediating paths and effect values were: mother’s rejection → self-concept → subjective well-being (−1.0476); mother’s rejection → emotional intelligence → subjective well-being (−0.0338); and mother’s rejection → self-concept → emotional intelligence → subjective well-being (−0.5710). The path of mother’s rejection → emotional intelligence → subjective well-being contained 0 in the 95% confidence interval, indicating that the indirect effect of the mediating variable of emotional intelligence was not significant. The confidence intervals for the other two paths did not contain 0, indicating that the indirect effects of both mediating variables were significant (Table 10).

**Table 10:** An analysis of the chain-mediated effects of self-concept and emotional intelligence on the relationship between mother’s rejection and subjective well-being.

	Effect value	BootSE	BootLLCI	BootULCI
Total effect	−2.1953	0.1765	−2.5414	−1.8492
Direct effect	−0.5429	0.125	−0.7881	−0.2977
Mediating effect	−1.6524	0.1421	−1.94	−1.3842
MR-SC-SWB	−1.0476	0.0937	−1.2415	−0.8777
MF-EI-SWB	−0.0338	0.0619	−0.1535	0.092
MR-SC-EI-SWB	−0.571	0.0591	−0.6925	−0.4599

MR = mother’s rejection; SC=Self-concept; SWB=Subjective well-being; EI = Emotional intelligence.

**Figure 4:**
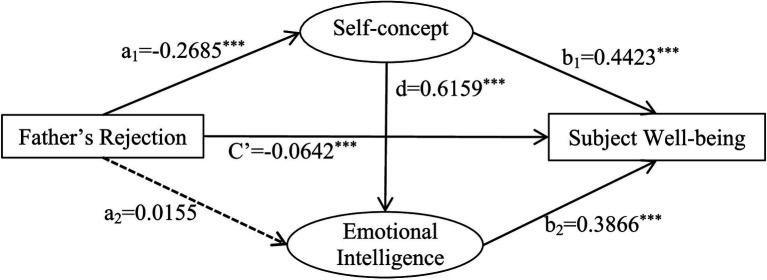
A chain-mediated model of fathers’ rejection, self-concept, emotional intelligence and subjective well-being.

**Figure 5:**
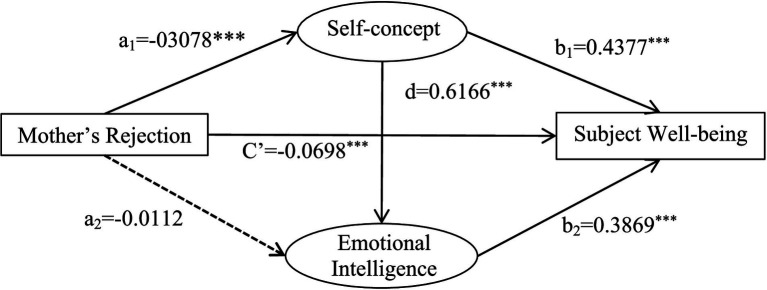
A chain-mediated model of mothers’ rejection, self-concept, emotional intelligence and subjective well-being.

A chain mediation model was established with the rejection parenting style of fathers and mothers as independent variables, self-concept, emotional intelligence as intermediate variables, and subjective well-being as the dependent variable (Figures 4, 5).

#### 3.2.3 Mediating role of self-concept and emotional intelligence between the overprotective parenting style and subjective well-being

Regression analysis showed that parental overprotection significantly negatively predicted self-concept, emotional intelligence, and subjective well-being. Self-concept significantly positively predicted emotional intelligence and subjective well-being, and emotional intelligence significantly positively predicted subjective well-being (Tables 11, 12).

**Table 11:** Regression analysis of the relationship between father’s overprotection variables.

Regression equation		Global FIT index		Significance of regression coefficient	
Result variable	Predictive variables	*R* ^2^	*F*	*β*	*t*
Self-concept	Gender	0.0543	34.1994	0.0285	1.2278
	Age			0.0548	2.3807
	Father’s overprotection			−0.2203	−9.486
Emotional intelligence	Gender	0.4013	299.3284	0.081	4.3791
	Age			0.0376	2.0518
	Self-concept			0.6168	32.7644
	Father’s overprotection			−0.0147	−0.7742
Subjective well-being	Gender	0.5802	493.4379	−0.0196	−1.2559
	Age			−0.0185	−1.2019
	Emotional intelligence			0.3877	19.5631
	Self-concept			0.4555	22.8281
	Father’s overprotection			−0.0147	−0.9238

**Table 12:** Regression analysis of the relationship between father’s overprotection variables.

Regression equation		Global FIT Index		Significance of regression coefficient	
Result variable	Predictive variables	*R* ^2^	*F*	*β*	*t*
Self-concept	Gender	0.0791	51.1656	0.027	1.1797
	Age			0.0542	2.3864
	Mother’s overprotection			−0.271	−11.8551
Emotional intelligence	Gender	0.4012	299.1516	0.082	4.4452
	Age			0.0377	2.0538
	Self-concept			0.6179	32.3819
	Mother’s overprotection			−0.008	−0.4196
Subjective well-being	Gender	0.581	495.118	−0.0211	−1.1976
	Age			−0.0184	−1.3622
	Emotional intelligence			0.3877	19.5809
	Self-concept			0.4497	22.3601
	Mother’s overprotection			−0.0335	−2.0932

Self-concept and emotional intelligence played a mediating role in overprotective parenting style and subjective well-being. The total mediation effect was −0.0828. The mediating pathways and effect values were: father’ overprotection → self-concept → subjective well-being (−0.5665); father’s overprotection → emotional intelligence → subjective well-being (−0.0321); and father’s overprotection → self-concept → emotional intelligence → subjective well-being (−0.2974). The path of father’s overprotection → emotional intelligence → subjective well-being contained 0 in the 95% confidence interval, which indicated that the indirect effect of emotional intelligence as a mediator variable was not significant. The other two paths did not contain 0 in the 95% confidence intervals, indicating that the indirect effects of both mediating variables were significant (Table 13).

**Table 13:** An analysis of the chain-mediated effects of self-concept and emotional intelligence on the relationship between father’s overprotection and subjective well-being.

	Effect value	BootSE	BootLLCI	BootULCI
Total effect	−0.9787	0.1325	−1.2386	−0.7189
Direct effect	−0.0828	0.0896	−0.2585	0.0929
Mediating effect	−0.896	0.1057	−1.1063	−0.6875
FO-SC-SWB	−0.5665	0.0671	−0.7008	−0.4368
FO-EI- SWB	−0.0321	0.0426	−0.1181	0.0505
FO-SC-EI-SWB	−0.2974	0.0394	−0.3797	−0.2241

FO=Father’s overprotection; SC=Self-concept; SWB=Subjective well-being; EI = Emotional intelligence.

Self-concept and emotional intelligence played a mediating role in mother’s overprotective parenting style and subjective well-being. The total mediating effect was −0.9861. The mediating pathways and effect values were: mother’s overprotection → self-concept → subjective well-being (−0.6329); mother’s overprotection → emotional intelligence → subjective well-being (−0.0162); and mother’s overprotection → self-concept → emotional intelligence → subjective well-being (−0.3371). The path of mother’s overprotection → emotional intelligence → subjective well-being contained 0 in 95% confidence interval, which indicated that the indirect effect of emotional intelligence as a mediator variable was not significant. The confidence intervals of other two paths did not contain 0, indicating that the indirect effects of both mediating variables were significant (Table 14).

**Table 14:** An analysis of the chain-mediated effects of self-concept and emotional intelligence on the relationship between mother’s overprotection and subjective well-being.

	Effect value	BootSE	BootLLCI	BootULCI
Total effect	−1.1603	0.1203	−1.3963	−0.9243
Direct effect	−0.1742	0.0832	−0.3374	−0.011
Mediating effect	−0.9861	0.0958	−1.1722	−0.7963
MO-SC-SWB	−0.6329	0.0635	−0.7591	−0.5095
MO-EI- SWB	−0.0162	0.0386	−0.094	0.06
MO-SC-EI-SWB	−0.3371	0.038	−0.4125	−0.2642

MO = Mother’s overprotection; SC=Self-concept; SWB=Subjective well-being; EI = Emotional intelligence.

**Figure 6:**
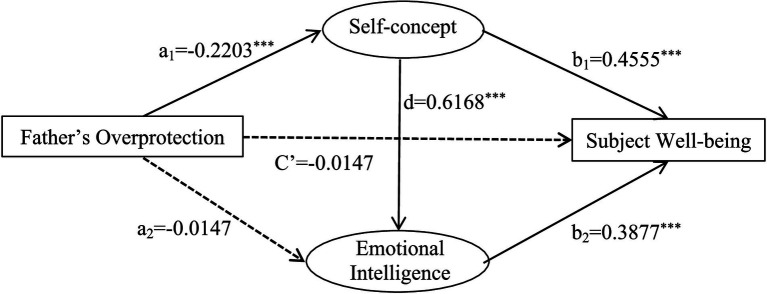
A chain-mediated model of father’s overprotection, self-concept, emotional intelligence and subjective well-being.

**Figure 7:**
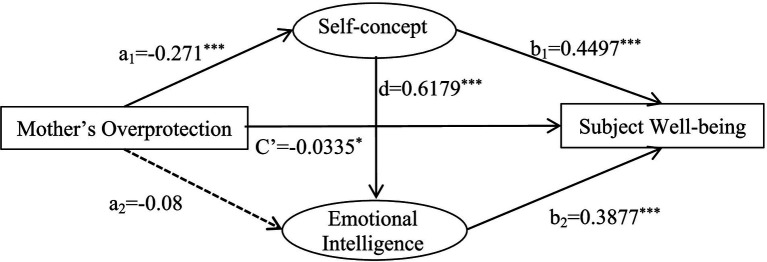
A chain-mediated model of mother’s overprotection, self-concept, emotional intelligence and subjective well-being.

A chain mediation model was established with father’s and mother’s overprotective parenting style as independent variables, self-concept, emotional intelligence as intermediate variables and subjective well-being as the dependent variable (Figures 6, 7).

## 4 Discussion

### 4.1 Main findings

#### 4.1.1 Direct impact of parenting styles on subjective well-being

This study examined the influence mechanism of parenting styles on the subjective well-being of primary school students by establishing a multiple mediation effect model. Overall, this study found that parenting styles had a significant direct impact on the subjective well-being of primary school students, which supported H1. Parents’ emotional warmth positively affected students’ subjective well-being, whereas rejecting and overprotective parenting styles negatively affected subjective well-being. The results of this study were consistent with many previous studies (Furnham and Cheng, 2000; Cheng et al., 2004; Lari, 2023). This study verified that parenting styles directly impacted the subjective well-being of primary school students.

#### 4.1.2 Mediating roles of self-concept and emotional intelligence

This study found that parenting styles influenced students’ subjective well-being through two mediating factors, self-concept and emotional intelligence. This supported H2 and H3. Parents’ emotional warmth affected subjective well-being through the mediating roles of self-concept and emotional intelligence. However, parental rejection and overprotection did not influence subjective well-being through the mediator of emotional intelligence, but rather through self-concept, via emotional intelligence.

On one hand, parenting styles influenced students’ subjective well-being through the mediating effect of self-concept. Parental emotional warmth positively affected self-concept. This may prompt primary school students to form a positive self-concept to overcome or negate adverse environmental and risk factors that may hinder healthy growth, thereby improving students’ subjective well-being. The rejection and overprotection parenting styles negatively affected self-concept. This may mean primary school students experienced self-ambiguity and self-uncertainty, which reduced their positive emotional experience, thereby potentially giving rise to internal psychological problems, self-defense, and perfectionist tendencies, and reducing students’ subjective well-being (Ezgi et al., 2018).

On the other hand, parenting styles affected students’ subjective well-being through the mediating role of emotional intelligence. This study concluded that emotional warmth positively affected emotional intelligence. Warmth, understanding and caring behavior shown by parents means students are more active in feeling, understanding, expressing and regulating emotions, thereby promoting development of individual emotional intelligence. However, the mediating effects of parental rejection and overprotection on emotional intelligence were not significant. Examination of these relational mechanisms showed father’s overprotection had no direct effect on subjective well-being under the mediation of emotional intelligence, but rather acted by influencing primary school students’ self-concept, which in turn affected subjective well-being. In contrast, a mother’s overprotective parenting style affected the development of children’s subjective well-being both directly and indirectly by affecting self-concept. Fathers’ overprotection makes it difficult for children to form a complete self-concept, which leads to problems in their perception of themselves, and therefore affects the formation of subjective well-being.

Additionally, this study found that parenting styles positively affected students’ well-being through self-concept via multiple mediating effects of emotional intelligence. This result supported H4 and also reveals another way that parenting styles indirectly affected subjective well-being. Previous research has shown a positive correlation between high emotional intelligence and self-concept (Argyriou et al., 2016; Mortazavizadeh et al., 2022). A healthy and well-balanced parenting style helps students develop higher levels of self-awareness; people with higher self-concept clarity develop precise and accurate perceptions of their differences from others, which allows them to develop emotional processing skills, high levels of emotional intelligence, and achieve high subjective well-being (Calero et al., 2018; Martínez-Monteagudo et al., 2021). The combination of self-concept and emotional intelligence ultimately determines the impact on subjective well-being, further confirming the close relationship between self-concept and emotional intelligence.

### 4.2 Implications of this research

This study explored the mediating variables that may affect primary school students’ subjective well-being, self-concept, and emotional intelligence, and confirmed the existence of a mediating role between parenting styles and subjective well-being. In particular, overprotective fathers played a fully mediating role, which suggested that parenting styles, through their influence on children’s self-concept and emotional intelligence, ultimately impacted their subjective well-being.

Based on an examination of real samples, the present study encourages parents to adopt emotionally warm parenting styles. Fathers should encourage their children to try new things as much as possible to enhance their children’s self-concept and emotional intelligence. This study also provides a scientific basis for family education, which is conducive to the promotion of family harmony and children’s healthy development.

### 4.3 Further research

This study had some limitations, and subsequent researchers can consider the following aspects. First, the sample size could be enlarged. This study only selected a sample from 10 schools in Hangzhou City, Zhejiang Province, China. However, the educational background of China differs from that in other countries, and there are disparities in the level of education and culture between regions. Second, this study only used a single way (questionnaires) to collect data. More data collection methods could be used in a similar study (e.g., family observation). Multiple methods could also be used to make up for the shortcomings of simple data analysis. Finally, dynamic observation can be performed. This study was a static study, and parents’ parenting styles and students’ growth are constantly changing, and dynamic observations of parents and students could be conducted to ensure the real-time nature of the study.

## 5 Conclusion

This study found that parental emotional warmth was positively related to primary school students’ subjective well-being, whereas parental rejection and overprotection were negatively related to subjective well-being. There were chained multiple mediating effects of self-concept and emotional intelligence in the relationship between parenting styles and subjective well-being. This study empirically supports the idea that positive parenting styles have a positive effect on primary school students’ subjective well-being. In addition, the mediating effects of self-concept and emotional intelligence expanded and enriched understanding of the relational mechanisms between parenting styles and primary school students’ subjective well-being.

The results of this study emphasize the importance of self-concept and emotional intelligence in the relationship between parenting styles and students’ subjective well-being. For Chinese parents, especially fathers, it is important to adopt an emotional warm parenting style when communicating with their children and try to avoid an overprotective parenting style. Furthermore, fathers should minimize excessive control over children’s behaviors. Based on the above findings, this study also suggests that special attention should be paid to the development of self-concept and emotional intelligence in primary school students, as these factors have a significant impact on promoting their subjective well-being.

## Data Availability

The raw data supporting the conclusions of this article will be made available by the authors, without undue reservation.
